# Prognostic and Clinicopathological Value of* PINX1* in Various Human Tumors: A Meta-Analysis

**DOI:** 10.1155/2018/4621015

**Published:** 2018-07-16

**Authors:** Hao Liang, Zhiyong Xiong, Ying Li, Weihao Kong, Zhicheng Yao, Ruixi Li, Meihai Deng, Kunpeng Hu

**Affiliations:** ^1^Department of Hepatobiliary Surgery, The Third Affiliated Hospital, Sun Yat-sen University, Guangzhou 510000, China; ^2^Department of General Surgery, The Third Affiliated Hospital, Sun Yat-sen University, Guangzhou 510000, China; ^3^Center for Reproductive Medicine, Department of Gynecology and Obstetrics, Nanfang Hospital, Southern Medical University, Guangzhou 510000, China; ^4^Department of Emergency Surgery, The First Affiliated Hospital, Anhui Medical University, Hefei 230022, China

## Abstract

*PINX1 *(Pin2/TRF1 interacting protein X1, an intrinsic telomerase inhibitor and putative tumor suppressor gene) may represent a novel prognostic tumor biomarker. However, the results of previous studies are inconsistent and the prognostic value of* PINX1* remains controversial. Therefore, we conducted a meta-analysis to determine whether* PINX1* expression is associated with overall survival (OS), disease-specific survival (DSS), disease-free survival (DFS), recurrence-free survival (RFS), and clinicopathological characteristics in patients with malignant tumors. A systematic search was performed in the PubMed, Web of Science, and Embase databases in April 2018. Quality assessment was performed according to the modified Newcastle-Ottawa Scale. Pooled odds ratios (ORs) and hazard ratios (HRs) with 95.0% confidence intervals (CIs) were calculated to determine the relationship between* PINX1* expression and OS, DSS, DFS/RFS, and clinicopathological characteristics. Due to the heterogeneity across the included studies, subgroup and sensitivity analyses were performed. Fixed-effects models were used when the heterogeneity was not significant and random-effects models were used when the heterogeneity was significant. Fourteen studies of 16 cohorts including 2,624 patients were enrolled. Low* PINX1* expression was associated with poor OS (HR: 1.51, 95.0% CI: 1.03–2.20;* P *= 0.035) and DFS/RFS (HR: 1.78, 95.0% CI: 1.28–2.47;* P *= 0.001) but not DSS (HR: 0.80, 95.0% CI: 0.38–1.67;* P *= 0.548). Low* PINX1* expression was also associated with lymphatic invasion (OR: 2.23, 95.0% CI: 1.35–3.70;* P *= 0.002) and advanced tumor-node-metastasis stage (OR: 2.43, 95.0% CI: 1.29–4.57;* P *= 0.006). No significant associations were observed between low* PINX1* expression and sex, depth of invasion, grade of differentiation, and distant metastasis. Low* PINX1* expression was associated with poor OS and DFS/RFS and lymphatic invasion and advanced tumor-node-metastasis stage, suggesting that* PINX1* expression may be a useful predictor of prognosis in patients with malignant tumors.

## 1. Introduction

A hallmark of cancer cells is their limitless division and maintenance of stable telomere lengths through activation of specific telomere maintenance mechanisms [1]. Telomeres, regions of repetitive DNA at the end of a chromosome, maintain chromosome integrity and genome stability, preventing double-strand breaks that can lead to aberrant chromosomal rearrangements [2–4]. Aberrations in telomere biology play a pivotal role in oncogenesis and in maintaining the potential of cancer cells to divide infinitely. In the last 15 years, researchers have proposed strategies to target telomerase or telomeric structure as a prospective approach to cancer therapy.

*PINX1 *(Pin2/TRF1 interacting protein X1) is a newly cloned gene mapped to chromosome 8p23.1. It encodes a 45-kDa nucleolar protein containing 328 amino acids that suppresses telomerase activity, resulting in telomere shortening [5, 6]. PinX1 is an interacting protein of telomeric repeat factor 1 (TRF1) that maintains telomere integrity by regulating TRF1 stability. Human telomerase reverse transcriptase may act as a regulator of TRF1 homeostasis in a PinX1-dependent manner [7, 8].

*PINX1* is a haploinsufficiency telomerase inhibitor and putative tumor suppressor gene [7, 9, 10]. The most frequently deleted region correlates with tumorigenicity and tumor invasion, migration, and differentiation. Several recent studies [10–19] have shown that low* PINX1* expression is associated with poor survival and different clinicopathological characteristics in various types of cancer, including colorectal cancer, ovarian carcinoma, breast cancer, non-small cell lung cancer, bladder urothelial carcinoma, renal cell carcinoma, and prostate cancer. However, conflicting results exist for some types of malignant tumors, including glioma, esophageal squamous cell carcinoma, and cervical squamous cell carcinoma [20–22]. Therefore, the precise prognostic role of* PINX1 *in malignant tumors remains controversial.

Therefore, we conducted this first meta-analysis to determine the prognostic and clinicopathological value of low* PINX1 *expression in patients with various types of malignant tumors.

## 2. Materials and Methods

### 2.1. Literature Search Strategy

A systematic search was performed in the PubMed, Web of Science, and Embase databases in April 2018. Search terms included “PinX1” OR “PinX1 protein” OR “PIN2-interacting protein 1” OR “liver putative tumor suppressor” AND “cancer” OR “tumor” OR “neoplasm” OR “carcinoma” OR “malignancy”. The published language was limited to English. A manual search was conducted to identify all potentially eligible studies, including references cited in original studies.

### 2.2. Inclusion and Exclusion Criteria

Eligible publications for inclusion met the following criteria: (1) studies reporting the relationship between* PINX1* expression and overall survival (OS), disease-specific survival (DSS), and disease-free survival(DFS)/recurrence-free survival(RFS) in patients with malignant tumors; (2) related clinicopathological data available; (3) studies published in English; (4) two groups of patients established based on* PINX1* expression; and (5) sufficient information or data available to calculate odds ratios (ORs) or hazard ratios (HRs) with 95.0% confidence intervals (CIs). The exclusion criteria included the following: (1) comments, letters, abstracts, reviews, case reports, meta-analyses, and duplicate studies; (2) studies with insufficient information or data to calculate ORs or HRs with 95.0% CIs; (3) studies conducted only on human cell lines or animals.

### 2.3. Data Extraction and Quality Assessment

Two independent investigators reviewed all eligible articles and carefully extracted the data. A third investigator was responsible for reconciling disagreements when the results were inconsistent. The extracted information included the name of the first author and year of publication, country, tumor type, study design, number of cases, mean age of the patients, method of analyzing* PINX1* expression, cutoff value, number of cases expressing* PINX1*, outcome measures, and follow-up time. When prognostic data from univariate and multivariate analyses were available, only the latter were extracted because of the accuracy in accounting for confounding factors. Only when Kaplan-Meier curves were presented for OS, DSS, and DFS/RFS without HRs or 95.0% CIs was the survival data extracted using Engauge Digitizer software (version 4.1, available to download from http://digitizer.sourceforge.net/). Two independent investigators assessed the quality of each study according to the modified Newcastle-Ottawa Scale (NOS) [24]. NOS scores were calculated based on selection, comparability, and ascertainment of the outcome of interest. Studies with NOS scores of ≥6 were considered high-quality studies [25].

### 2.4. Statistical Analyses

All statistical analyses were conducted using Stata software (version 12.0; Stata Corporation, College Station, TX, USA). To determine whether* PINX1* expression was associated with tumor prognosis, HRs and 95.0% CIs were calculated for the quantitative aggregation of survival data. When included studies did not provide HRs and corresponding 95.0% CIs, methods developed by Parmar [26], Williamson [27], and Tierney [28] were applied to extract data from survival curves. Regarding the relationship between* PINX1 *expression and the clinicopathological characteristics of malignant tumors, ORs and 95.0% CIs were used to calculate the pooled results. A* P *< 0.05 was considered statistically significant. The heterogeneity of the included studies was detected using Chi-square tests and* I*^2^ statistics.* I*^2^ > 50.0% with* P *< 0.05 indicated statistically significant heterogeneity. Pooled ORs and HRs were calculated using fixed-effects or random-effects models. Publication bias was assessed visually (by evaluating the symmetry of funnel plots) and formally (using Egger's and Begg's tests). Funnel plot asymmetry and* P *< 0.05 indicated significant publication bias. Sensitivity analysis was used to confirm the reliability and stability of the meta-analysis.

## 3. Results

### 3.1. Literature Search

**Figure 1:**
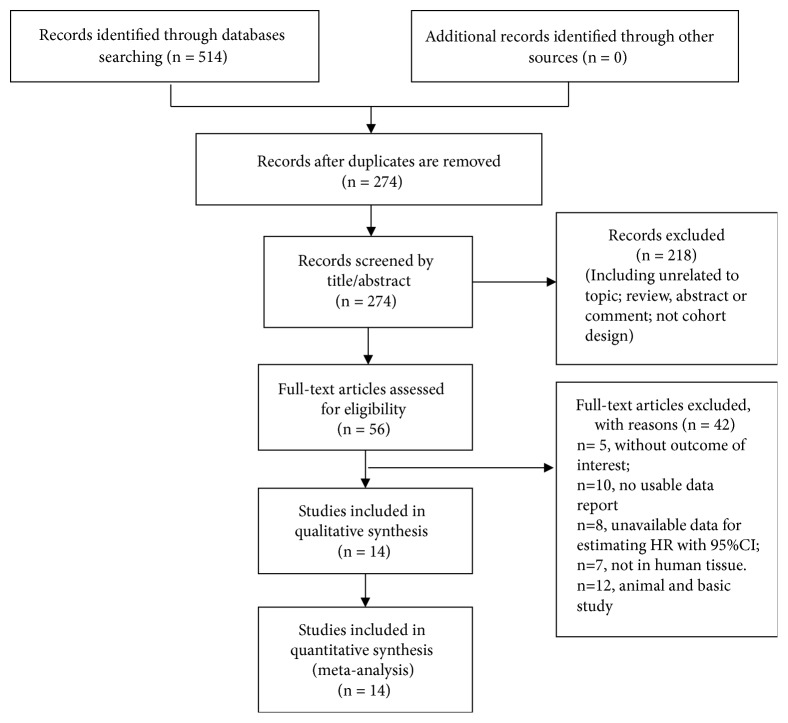
Flow diagram of study selection strategy. CI, confidence interval; HR, hazard ratio.

In the initial search, 514 studies were aggregated and analyzed from the PubMed, Web of Science, and Embase databases. Duplicates (*n *= 240) were removed. The titles and abstracts were screened, leaving 56 studies for detailed analysis. After meticulously reviewing the full texts, 42 studies were excluded. Finally, 14 eligible studies containing 2,624 patients [10–23] were included in the meta-analysis. A flow chart of the screening process is shown in Figure 1.

### 3.2. Study Characteristics

A total of 14 studies with 16 cohorts were extracted. The baseline characteristics of the studies are summarized in Table 1. Finally, 2,624 patients with malignant tumors were enrolled in the subsequent analysis. The included studies (published between 2008 and 2017) were all conducted in China. The sample size across all eligible studies ranged from 40 to 583. All of the included studies were retrospective analyses that were published in English. Qian et al. [21] and Tian et al. [18] each conducted 2 studies. Therefore, we labeled them as Qian 1 and Qian 2 and Tian 1 and Tian 2, respectively.* PINX1* expression in tissue specimens was quantified using immunohistochemistry (IHC) or IHC and tissue microarray (TMA). Cutoff values were determined using the staining index or percentage of stained cells. Two studies [12, 13] reported on colorectal cancer, 2 [14, 15] reported on breast cancer, 2 [18, 22] reported on non-small cell lung cancer, and 1 each reported on glioma [20], ovarian carcinoma [11], renal cell carcinoma[10], bladder urothelial carcinoma [16], gastric cancer [23], esophagus cancer [21], prostate cancer [17], and cervical carcinoma [22]. Ten studies with 11 cohorts [10–13, 15, 16, 18–20, 22] reported on OS, 4 studies with 5 cohorts [10, 15, 20, 21] reported on DSS, and 3 studies [12, 13, 19] reported on DFS. Because only 1 study [16] reported on RFS, DFS and RFS were combined to calculate the pooled HR and 95.0% CI. The mean NOS score of the included studies was 6.2 (range, 5.0–7.0), suggesting that the quality of the included studies was adequate (Table 2).

### 3.3. Associations Between* PINX1 *Expression and OS

**Figure 2:**
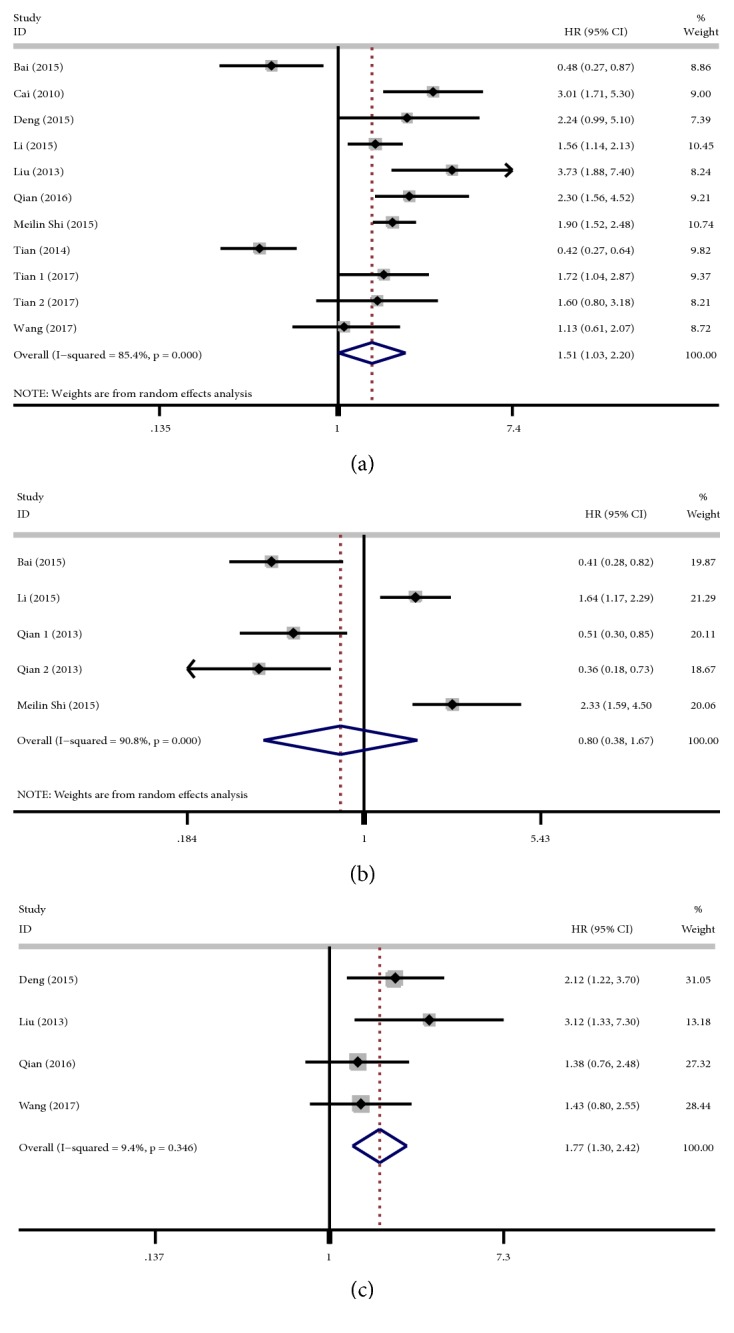
Forest plot of combined analyses associated with* PINX1 *expression. (a) The forest plot for the association between* PINX1* expression and overall survival (OS). Low* PINX1* expression was associated with poorer OS (HR: 1.51, 95.0% CI: 1.03–2.20;* P *= 0.035). (b) The forest plot for the association between* PINX1* expression and disease-specific survival (DSS).* PINX1* expression was not associated with DSS (HR: 0.80, 95.0% CI: 0.38–1.67;* P *= 0.548). (c) The forest plot for the association between* PINX1* expression and disease-free/recurrence-free survival (DFS/RFS). Low* PINX1* expression was associated with poorer DFS/RFS (HR: 1.77, 95.0% CI: 1.30–2.42;* P* = 0.001). CI, confidence interval; HR, hazard ratio.

OS was analyzed in 10 studies with 11 cohorts [10–13, 15, 16, 18–20, 22]. Due to the existence of heterogeneity (*I*^2^ = 85.4%,* P *< 0.001), a random-effects model was used to calculate the pooled HR. Our meta-analysis indicated that low* PINX1* expression was associated with significantly poorer OS (HR: 1.51, 95.0% CI: 1.03–2.20;* P *= 0.035) (Figure 2(a)).

Owing to severe heterogeneity, subgroup analyses stratified by tumor type, sample size, test method, cutoff value, type of analysis, and NOS score were conducted to investigate the association between* PINX1* expression and OS in patients with various types of malignant tumors (Table 3). In a subgroup analysis of tumor type, low* PINX1* expression was associated with significantly poorer OS for colorectal cancer (HR: 2.28, 95.0% CI: 1.46–3.56;* P *< 0.001) and non-small cell lung cancer (HR: 1.48, 95.0% CI: 1.06–2.08;* P *= 0.023). However, no significant associations were observed between low* PINX1 *expression and other types of malignant tumors (HR: 1.36, 95.0% CI: 0.74–2.50;* P *= 0.322). Moreover, the prognostic significance of low* PINX1 *expression for OS was poorer with respect to sample size (> 150) (HR: 1.70, 95.0% CI: 1.15–2.50;* P *= 0.007) and test method (IHC +TMA) (HR: 1.72, 95.0% CI: 1.05–2.84;* P *= 0.032). No significant differences were observed in any other subgroup analyses.

### 3.4. Associations Between* PINX1 *Expression and DSS

Four studies with 5 cohorts [10, 15, 20, 21] reported associations between* PINX1* expression and DSS. The results of the meta-analysis using a random-effects model (*I*^2^ = 90.8%,* P *< 0.001) showed that low* PINX1* expression was not associated with significantly poorer DSS (HR: 0.80, 95.0% CI: 0.38–1.67;* P *= 0.548) (Figure 2(b)).

### 3.5. Associations Between* PINX1 *Expression and DFS/RFS

Four studies [12, 13, 16, 19] reported associations between* PINX1* expression and DFS/RFS. A fixed-effects model was used to calculate the pooled HR and 95.0% CI because no significant heterogeneity was observed (*I*^2^ = 9.4%,* P *= 0.346). The results of the meta-analysis showed that low* PINX1* expression was associated with significantly poorer DFS/RFS (HR: 1.77, 95.0% CI: 1.30–2.42;* P *= 0.001) (Figure 2(c)).

### 3.6. Publication Bias and Sensitivity Analysis

**Figure 3:**
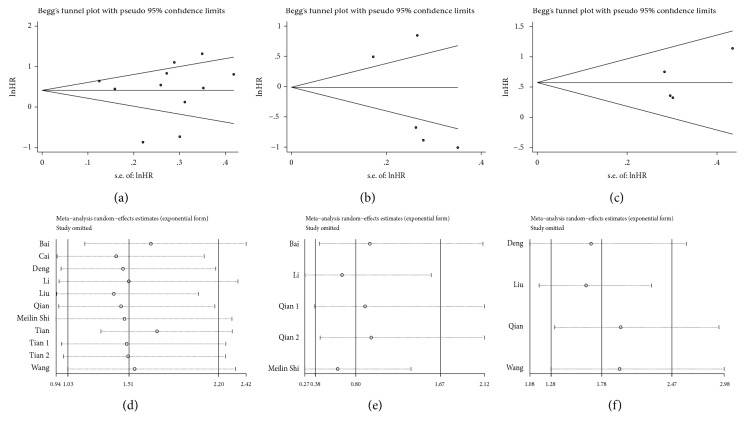
Begg's funnel plot of publication bias for (a) overall survival, (b) disease-specific survival, and (c) disease-free/recurrence-free survival and sensitivity analysis for (d) overall survival, (e) disease-specific survival, and (f) disease-free/recurrence-free survival.

Begg's funnel plot (Figures 3(a)–3(c)) and Egger's test were used to assess the potential publication bias of the OS, DSS, and DFS/RFS studies included in this meta-analysis. The symmetrical shape of the funnel plots and the* P *values from Begg's and Egger's tests indicated that there was no significant publication bias for OS, DSS, and DFS/RFS (*P *= 0.755 and* P *= 0.914,* P *= 0.221 and* P *= 0.213, and* P *= 1.000 and* P *= 0.336, respectively).

To determine the influence of each study on the pooled HRs for OS, DSS, and DFS/RFS and to verify the robustness of our results, sensitivity analysis was performed by omitting one study at a time and calculating the pooled HRs for the remaining studies. The results of the sensitivity analysis indicated that no significant effect on pooled HRs was observed after excluding any single study, suggesting that the results of this meta-analysis were relatively robust (Figures 3(d)–3(f)).

### 3.7. Associations Between* PINX1 *Expression and Clinicopathological Characteristics

Nine [10, 12, 13, 16, 18–21, 23], 11 [10–19, 21], 12 [10–19, 21, 23], 11 [11–18, 21–23], 10 [10–14, 17–20, 23], and 5 [11, 17–19, 21] studies reported associations between* PINX1* expression and sex, depth of invasion, lymph node metastasis, tumor differentiation, tumor-node-metastasis (TNM) stage, and distant metastasis, respectively. Associations between low* PINX1* expression and clinicopathological characteristics are summarized in Table 4. The results of the pooled analysis showed that low* PINX1* expression was associated with lymph node metastasis (OR: 2.23, 95.0% CI: 1.35–3.70;* P *= 0.002) and advanced TNM stage (OR: 2.43, 95.0% CI: 1.29–4.57;* P *= 0.006). However, no significant associations were observed between low* PINX1 *expression and sex (OR: 0.84, 95.0% CI: 0.65–1.09;* P *= 0.168), depth of invasion (OR: 1.57, 95.0% CI: 0.88–2.78;* P *= 0.126), grade of differentiation (OR: 1.35, 95.0% CI: 0.91–2.02;* P *= 0.140), and distant metastasis (OR: 2.64, 95.0% CI: 0.87–8.01;* P *= 0.087).

## 4. Discussion

The human* PINX1 *gene comprises 7 exons. It maps to a region that is frequently associated with loss of heterozygosity in cancer [29–33]. PinX1 is an interacting protein of TRF1 that regulates TRF1 stability to maintain telomere integrity [7, 8]. The human* PINX1 *gene is a telomerase inhibitor and putative tumor suppressor gene that inhibits telomerase activity and shortens the length of telomeres to suppress tumor growth [34]. In addition, low* PINX1* expression has been shown to affect chemoradiotherapy sensitivity in cancer cells [21, 22, 35]. The association between* PINX1 *dysregulation and carcinogenesis has been described in several reports. However, low* PINX1* expression plays various roles in tumor progression and its prognostic value in patients with malignant tumors remains controversial. To date, no meta-analyses have been conducted to determine the prognostic value of low* PINX1 *expression in patients with malignant tumors. Therefore, we conducted a meta-analysis of 14 studies containing 2,624 patients to determine the prognostic value of* PINX1* expression for survival in patients with malignant tumors. Furthermore, this is the first meta-analysis to determine whether* PINX1* expression is associated with patient outcomes.

The pooled results of the meta-analysis showed that low* PINX1 *expression was associated with significantly poorer OS and DFS/RFS, but not DSS, in patients with malignant tumors. To investigate the cause of heterogeneity, subgroup analyses were performed according to the tumor type, sample size, test method, cutoff value, type of analysis, and NOS score. The results of the subgroup analysis showed that low* PINX1* expression was associated with significantly poorer OS in colorectal cancer, non-small cell lung cancer, sample size (> 150), and test method (IHC + TMA). Therefore,* PINX1 *expression is a potential biomarker of prognostic significance for OS and DFS/RFS in colorectal cancer and non-small cell lung cancer. However, studies of other malignant tumors are limited and not all of the included studies involved all types of malignant tumors. Therefore, more eligible studies are needed to validate the prognostic value of* PINX1 *expression in other types of malignant tumors. Sample sizes and test methods caused heterogeneity in the results. Therefore, the standard sample size and test method should be verified based on further eligible studies.

The pooled results for OS and DFS/RFS showed that low* PINX1 *expression was associated with poor prognosis, suggesting that* PINX1 *may function as a tumor suppressor gene in malignant tumors. However, among the 14 studies included in this meta-analysis, 3 [20–22] reported on cervical squamous cell carcinoma, esophageal squamous cell carcinoma, and glioma where* PINX1* expression has been reported to have an opposite prognostic effect compared to the pooled outcome. Meanwhile, high* PINX1* expression has been reported to be associated with response to chemoradiotherapy. It has also been reported to be an independent predictor of poor survival in cervical and esophageal squamous cell carcinomas [21, 22]. The potential reasons are as follows. First, only a single study reported the prognostic value of* PINX1* expression in each of the following: cervical squamous cell carcinoma, esophageal squamous cell carcinoma, and glioma, respectively [20–22]. The small sample size may have introduced bias. Moreover, the relationship between* PINX1* expression and response to chemoradiotherapy was considered a clinical parameter in cervical and esophageal squamous cell carcinomas, the mechanism of which has not been determined [21, 22]. Therefore, the results may have statistical flaws and larger sample sizes may be needed to address this bias. Hence, we conducted a meta-analysis to increase the sample size and to reduce bias in assessing the prognostic value of* PINX1 *expression in various types of malignant tumors. Second, the genetic background of* PINX1 *may be different in different types of tumors and the role of PinX1 in tumorigenesis complicated and may be tumor type-specific. Furthermore, the mechanism of PinX1 may change in response to chemoradiotherapy, which may influence the prognosis of patients with malignant tumors. Hence, the results of our meta-analysis should be interpreted with caution. Further eligible studies are needed to determine the prognostic value of* PINX1 *expression in various types of malignant tumors.

With respect to the clinicopathological characteristics, we showed that low* PINX1* expression was associated with lymph node metastasis and advanced TNM stage. This suggests that low* PINX1* expression is linked to enhanced invasion and migration capacity and reduced survival. Previous studies [10, 15, 16, 36, 37] have described the molecular mechanisms* in vitro*. PinX1 inhibits invasion, migration, and cell proliferation by suppressing the NF-*κ*B/MMP-9 signaling pathway and MMP-2 via NF-*κ*B-dependent transcription, as well as inhibiting telomerase activity via the p16/cyclin D1 pathway and the Mad1/c-Myc pathway and increasing the apoptotic index. Therefore, low* PINX1* expression promotes tumor proliferation, invasion, and migration. However, no significant differences were observed for sex, depth of invasion, grade of differentiation, and distant metastasis. Our findings suggest that PinX1 may facilitate the invasion and migration of malignant tumors. However, further studies are needed to verify the contradictory results and to confirm an association between* PINX1* expression and sex, depth of invasion, grade of differentiation, and distant metastasis.

This is the first meta-analysis to evaluate the prognostic value of* PINX1* expression in patients with malignant tumors. However, there are several limitations, which mean caution must be taken when interpreting the results. First, all of the included studies were published in English, which may have resulted in publication bias. Second, the included studies did not involve all types of malignant tumors (some types of malignant tumors were reported in only a single study), which suggests that more high-quality studies were necessary to accurately determine the prognostic value of* PINX1 *expression in patients with malignant tumors. Third, different biological characteristics and molecular mechanisms associated with different types of malignant tumors may generate potentially inconsistent findings. Fourth, some data were extracted from survival curves in the included studies which may be less reliable than data obtained directly. Finally, all of the patients included in this meta-analysis were Asian. Therefore, it is necessary to validate our findings in different populations.

## 5. Conclusions

In this meta-analysis, we showed that low* PINX1* expression was associated with significantly poorer OS, DFS/RFS, and clinicopathological characteristics in patients with various types of malignant tumors. We highlight the important role of PinX1 in malignant tumors and reveal the potential for* PINX1 *expression to serve as a biomarker for malignant tumors

## Figures and Tables

**Table 1:** Baseline characteristics of all of the studies included in the meta-analysis.

Study	Country	Type of malignancy	Study design	No. of patients	Mean age (years)	Test method	Cutoff value	No. of cases with PINX1 expression		Outcome measures	Median follow- up time (months)	NOS score
								Low	High			
Bai 2015 [20]	China	Glioma	R	583	47	IHC + TMA	Staining index^*∗*^ ≤4	217	366	OS^MA^ DSS^MA^	60	7
Cai 2010 [11]	China	Ovarian carcinoma	R	157	51	IHC + TMA	Stained cells <60.0%	53	104	OS^UA^	NA	6
Deng 2015 [12]	China	Colorectal cancer	R	83	58	IHC	Staining index^*∗*^ ≤4	36	47	OS^UA^ DFS^UA^	NA	6
Feng 2017 [14]	China	Breast cancer	R	59	50	IHC + TMA	Stained cells <62.5%	35	24	NA	NA	6
Li 2015 [10]	China	Renal cell carcinoma	R	353	NA	IHC + TMA	Staining index^*∗*^ ≤3	203	150	OS^MA^ DFS^MA^	60	5
Liu 2013 [16]	China	Bladder urothelial carcinoma	R	187	60	IHC + TMA	Stained cells <50.0%	83	104	OS^UA^ RFS^UA^	92	7
Ma 2008 [23]	China	Gastric cancer	R	90	NA	IHC	Staining index^*∗*^ ≤3	38	52	NA	NA	5
Qian 1 2013 [21]	China	Esophageal cancer	R	98	55	IHC	Stained cells <50.0%	40	58	DSS^MA^	NA	7
Qian 2 2013 [21]	China	Esophageal cancer	R	59	55	IHC	Stained cells <50.0%	26	33	DSS^MA^	NA	7
Qian 2016 [13]	China	Colorectal cancer	R	86	55	IHC	Stained cells <50.0%	52	34	OS^MA^ DFS^MA^	NA	7
Rong Shi 2014 [17]	China	Prostate cancer	R	40	67	IHC + TMA	Stained cells <60.0%	27	13	NA	NA	5
Meilin Shi 2015 [15]	China	Breast cancer	R	405	NA	IHC + TMA	Staining index^*∗*^ ≤3	212	193	OS^MA^ DSS^MA^	60	7
Tian 2014 [22]	China	Cervical carcinoma	R	122	46	IHC	Stained cells <50.0%	53	69	OS^UA^	NA	6
Tian 1 2017 [18]	China	Non-small cell lung cancer	R	93	56	IHC	Stained cells <65.0%	56	37	OS^UA^	NA	6
Tian 2 2017 [18]	China	Non-small cell lung cancer	R	51	56	IHC	Stained cells <65.0%	27	24	OS^UA^	NA	6
Wang 2017 [19]	China	Non-small cell lung cancer	R	158	61	IHC	Stained cells <50.0%	117	41	OS^UA^ DFS^UA^	NA	7

^*∗*^Staining index = staining intensity × proportion of immune-positive cells.

DFS, disease-free survival; DSS, disease-specific survival; IHC, immunohistochemistry; MA, multivariate analysis; NA, not available; No., number; NOS, Newcastle-Ottawa Quality Assessment Scale; OS, overall survival; R, retrospective; RFS, recurrence-free survival; TMA, tissue microarray; UA, univariate analysis.

**Table 2:** Newcastle-Ottawa quality assessment scale.

First Author	Year	Selection	Comparability	Outcome	Total
Bai [20]	2015	★★★	★	★★★	7
Cai [11]	2010	★	★★	★★★	6
Deng [12]	2015	★★	★★	★★	6
Feng [14]	2017	★	★★	★★★	6
Li [10]	2015	★★	★	★★	5
Liu [16]	2013	★★	★★	★★★	7
Ma [23]	2008	★★	★★	★	5
Qian 1 [21]	2013	★★	★★	★★★	7
Qian 2 [21]	2013	★★	★★	★★★	7
Qian [13]	2016	★★	★★	★★★	7
Rong Shi [17]	2014	★★	★★	★	5
Meilin Shi [15]	2015	★★	★★	★★★	7
Tian [22]	2014	★★	★★	★★	6
Tian 1 [18]	2017	★	★★	★★★	6
Tian 2 [18]	2017	★	★★	★★★	6
Wang [19]	2017	★★	★★	★★★	7

**Table 3:** Subgroup analysis of pooled HRs for overall survival in patients with low PINX1 expression.

Stratified analysis	No. of cohorts	No. of patients	Pooled HR (95.0% CI)	P value	Heterogeneity		
					*I* ^*2*^ * (%)*	*P value*	Model
Tumor type							
Colorectal cancer	2	169	2.28 (1.46 – 3.56)	<0.001	0.0	0.961	Fixed
Non-small cell lung cancer	3	302	1.48 (1.06 – 2.08)	0.023	0.0	0.567	Fixed
Other	6	1,807	1.36 (0.74 – 2.50)	0.931		<0.001	Random
Sample size							
≤150	4	435	1.23 (0.52 – 2.88)	0.635	94.5	<0.001	Random
>150	7	1,843	1.70 (1.15 – 2.50)	0.007	88.2	<0.001	Random
Test method							
IHC only	6	568	1.34 (0.73 – 2.46)	0.341	80.4	<0.001	Random
IHC + TMA	5	1,710	1.72 (1.05 – 2.84)	0.032	85	<0.001	Random
Cutoff value							
Staining index ≤4	4	1,424	0.30 (-0.22 – 0.82)	0.255	85.5	<0.001	Random
Stained cells <65.0%	7	854	0.49 (-0.12 – 1.10)	0.116	83.8	<0.001	Random
Type of analysis							
Univariate	7	851	1.61 (0.86 – 3.03)	0.136	87.8	<0.001	Random
Multivariate	4	1,427	1.40 (0.86 – 2.29)	0.178	87.3	<0.001	Random
NOS score							
≤6	6	859	1.48 (0.83 – 2.65)	0.186	84.7	<0.001	Random
>6	5	1,419	1.55 (0.87 – 2.74)	0.134	84.9	<0.001	Random

CI, confidence interval; HR, hazard ratio; IHC, immunohistochemistry; No., number; NOS, Newcastle-Ottawa Quality Assessment Scale; TMA, tissue microarray.

**Table 4:** Meta-analysis of low *PINX1 *expression and clinicopathological characteristics of malignant tumors.

Clinicopathological characteristic	No. of cohorts	OR(95.0% CI)	*P* value	Heterogeneity		
				*I* ^2^(%)	*P* value	Model
Sex (male *vs.* female)	9	0.84(0.65 –1.09)	0.168	23.1	0.238	Fixed
Depth of invasion (T3–4 *vs.* T1–2)	11	1.57(0.88 –2.78)	0.126	81.1	<0.001	Random
Lymph node metastasis (yes *vs.* no)	12	2.23(1.35 –3.70)	0.002	77.5	<0.001	Random
Tumor differentiation (poor *vs.* well)	11	1.35(0.91 –2.02)	0.140	59.7	0.006	Random
TNM stage (III–IV *vs.* I–II)	10	2.43(1.29 – 4.57)	0.006	83.6	<0.001	Random
Distant metastasis (yes *vs.* no)	5	2.64(0.87– 8.01)	0.087	85.1	<0.001	Random

CI, confidence interval; No., number; OR, odds ratio; TNM, tumor-node-metastasis.
